# Tim-3 Blockade Elicits Potent Anti-Multiple Myeloma Immunity of Natural Killer Cells

**DOI:** 10.3389/fonc.2022.739976

**Published:** 2022-02-25

**Authors:** Wen Jiang, Fanglin Li, Yang Jiang, Shengli Li, Xiaoli Liu, Yaqi Xu, Binggen Li, Xiaoli Feng, Chengyun Zheng

**Affiliations:** ^1^ Institute of Medical Sciences, The Second Hospital, Cheeloo College of Medicine, Shandong University, Jinan, China; ^2^ Department of Hematology, The Qilu Hospital, Cheeloo College of Medicine, Shandong University, Jinan, China; ^3^ Department of Hematology, The Second Hospital, Cheeloo College of Medicine, Institute of Biotherapy for Hematological Malignancies, Shandong University, Shandong University-Karolinska College Collaborative Laboratory for Stem Cell Research, Jinan, China; ^4^ Department of Hematology, Jining NO.1 People’s Hospital, Jining, China; ^5^ R&D Department, Weihai Zhengsheng Biotechnology Co., Ltd, Weihai, China; ^6^ Department of Clinical Laboratory, The Second Hospital, Cheeloo College of Medicine, Shandong University, Jinan, China

**Keywords:** natural killer cells, Tim-3, multiple myeloma, anti-tumor immunity, adaptive immunotherapy

## Abstract

Multiple myeloma (MM) is still an incurable plasma cell tumor. Natural killer (NK) cells are characterized by efficient anti-tumor activity, and their activity is one basis of cancer immunotherapeutic strategies. Tim-3, one of the immune checkpoint molecules, negatively regulates NK cell activity. To evaluate roles of the Tim-3 pathway blocking in the regulation of NK cell mediated- anti-MM activity *in vitro* and *in vivo*, anti-Tim-3 and/or anti-its ligand (HMGB1, CEACAM1 or Galetin-9) antibodies were applied respectively to block the Tim-3 pathway in the present study. Our results showed that Tim-3 was highly expressed on NK cells, in particular on *in vitro* expanded NK (exNK) cells. NK cells with Tim-3 blockade displayed a significantly higher degranulation and cytolytic activity against both human MM cell lines and primary MM cells, compared to the isotype control antibody-treated NK cells. The increased NK cell cytolytic activity by Tim-3 blocking was associated with up-regulation of cytotoxicity-related molecules, including perforin, granzyme B, TNF-α and IFN-γ. Ligand (HMGB1, CEACAM1 or Galetin-9) expression on MM cells was at different levels, and accordingly, the improvement in NK cell-mediated killing activity by different ligand blocking were also varying. Tim-3 blocking showed much more efficient enhancement of NK cell cytolytic activity than its ligand blockings. More importantly, exNK cells with Tim-3 blockade significantly inhibited MM tumor growth and prolonged the survival of MM-bearing NOD/SCID mice. Our results also showed that NK cells from peripheral blood and bone marrow of MM patients expressed much higher levels of Tim-3 than their counterparts from controls. Taken together, Tim-3 may be an important target molecule used for developing an antibody and/or NK cell based immunotherapeutic strategies for MM.

## Introduction

Multiple myeloma (MM) is a malignant plasma cell tumor, characterized by an expansion of monoclonal plasma cells in the bone marrow and increased monoclonal immunoglobulin in the plasma. The major clinical presentations of the disease include hypercalcemia, renal failure, anemia, and lytic bone destruction. MM accounts for about 13% of all hematological malignancies, and its incidence is increasing every year, and has become the second most common hematological tumor worldwide. Clinical outcomes of MM have been greatly improved during the past decade due to the introduction of novel therapies. Especially in recent years, the application of new drugs such as proteasome inhibitors and immunomodulatory drugs have significantly improved the treatment efficacy and prolonged survival of MM patients (1, 2). However, MM remains incurable (3). Recently, with the integration and development of immunology, molecular biology, and cell biology, the role and mechanism of immune cells in the oncogenesis has become a new direction in cancer research. In particular, the study of tumor microenvironmental immunity has revealed the role of immune cells in the occurrence and development of MM, which is of great significance for not only the development of new precise immune targets for the treatment of MM, but also the understanding of the function of the immune system in MM.

MM cells parasitize the bone marrow (BM). Cells such as monocytes, T lymphocytes, NK cells, and mesenchymal stem cells in BM microenvironment play an important role in regulating tumor cell proliferation, myeloma bone disease, and tumor cells escaping from the immune system (4–6). NK cells are a group of large granular lymphocytes and are key components of the innate immune system. Different from T cells, NK cells could exert their cytotoxic effects against targets without pre-sensitization process. Additionally, NK cells are also involved in adaptive immune response by cross-talking with other type of immune cells such as dendritic cells and monocytes. Thus, NK cells are very important immune cells in the body fighting against tumors and infections. NK cells of MM patients have a certain degree of functional defects, which are mainly manifested as a decrease in NK cell killing activity. Previous studies demonstrated that NK cells from the patients of the relapsed and advanced group exhibited significantly decreased activity (7–10). Low or defective NK cell function predicts poor prognosis for patients (11, 12). However, it is unclear which factors trigger defective NK cells in MM patients, and how NK cell killing activity can be restored.

Tim-3 is a member of the Tim family and belongs to the immunoglobulin superfamily (13). Tim-3 is an inhibitory regulating molecule in the immune response. The combination of Tim-3 and its ligands can promote lymphocyte apoptosis and induce immune tolerance. Many studies have shown that Tim-3 is a key immune checkpoint molecule for tumor-induced immune suppression (14). In both solid and hematological tumors, CD8+T cells expressing Tim-3 represent a severe decline of its function, and furthermore, Tim-3+CD8+T cells usually co-express PD-1 molecules (15). Tumor-infiltrating T lymphocytes in patients with non-small cell lung cancer express high levels of Tim-3 and blocking Tim-3 effectively restores the secretion of IFN-γ and TNF-α of CD8+ T cells (14).

Tim-3 is expressed in normal human peripheral blood NK cells. When Tim-3 in NK cells bind to its ligand Galectin-9, the function of NK cells, such as cytokine secretion ability and killing activity, is inhibited. Pires da Silva suggests that Tim-3 is a molecular marker of NK cell function exhaustion, which is mainly shown in the Tim-3+NK cells with the up-regulation of the inhibitory receptors (such as KIR2DL1 and KIR2DL3), while down-regulation of the activating receptors (such as NKG2D, NKp46, and DNMA-1). Their study showed that Tim-3+NK cells play a negative immune regulatory role in the occurrence and development of melanoma. Blocking expression of Tim-3 in NK cells can significantly improve the effect of NK cells on killing melanoma cells (16).

On this basis, we used the anti-Tim-3 antibody to neutralize Tim-3 expression in NK cells to explore its role in the regulation of NK cell cytotoxicity against MM. Here, we report that Tim-3 blockaded NK cells exerted efficacious cytolysis against MM cells, including both MM cell lines and primary MM cells. Further investigations demonstrated that blocking Tim-3 in NK cells significantly inhibited the growth of MM xenograft tumors and prolonged the survival of MM bearing mice. By measuring expression and effect of the Tim-3 ligand on MM cells, we identified the key molecules through which Tim-3 inhibits in the killing effect of NK cells on MM. These results provide a proof-of-concept for Tim-3 and NK cell combined MM immunotherapy.

## Methods

### Cell Lines and Cell Culture

The human MM cell lines RPMI8226 and MM.1S, and the human natural killer cell line NK-92 were obtained from ATCC (Manassas, VA, USA). RPMI8226 and MM.1S cells were cultured in RPMI-1640 medium (GIBCO/BRL, Grand Island, NY, USA) supplemented with 100 U/ml penicillin-streptomycin and 10% fetal bovine serum (FBS). The RPMI8226-Luc cell line was constructed by luciferase-encoding lentiviral infection. NK-92 cells were cultured in MEM-alpha medium (GIBCO/BRL, Grand Island, NY, USA) supplemented with 100 U/ml rhIL-2, 100 U/ml penicillin-streptomycin, 12.5% FBS, 12.5% horse serum, and 0.1 mM β-mercaptoethanol. The human NK cell line NKL was a gift from Professor Jin BQ (Department of Immunology, Fourth Military Medical University, Xi’an, PR China). NKL cells were cultured in complete RPMI-1640 medium containing (100 U/ml) rhIL-2. All cells were incubated at 37°C in a humidified incubator with 5% CO_2_. Cells in the logarithmic growth phase were selected for subsequent experiments.

### The exNK Cell Expansion System

Primary NK cells from healthy individuals were expanded by following the methods described in our preliminary work (9). Briefly, hPBMCs derived from healthy donors were stimulated with anti-CD16 mAb and a high concentration of IL-2. After culturing for 21-days, expanded NK (exNK) cells were harvested and analyzed for future study. Cells were maintained in CellGro^®^ DC medium containing 10% FBS, 100 U/ml penicillin/streptomycin, and 2 mM glutamine.

### Patients

This study was approved by the Human Ethics Research Committee of the Second Hospital of Shandong University (approval no. KYLL-2017(KJ) P-0106). Human BM samples were obtained from newly diagnosed or relapsed/refractory MM patients at the Second Hospital of Shandong University, Qilu Hospital of Shandong University, and the Affiliated Hospital of Jining Medical University. Age-matched healthy individuals and iron deficiency anemia patients with non-infectious, non-tumor, non-autoimmune diseases were recruited as controls.

### Isolation of Human Bone Marrow Mononuclear Cells (hBMMCs) and Human Peripheral Blood Mononuclear Cells (hPBMCs)

Human peripheral blood and bone marrow were obtained from MM patients or control donors. The hBMMCs and hPBMCs were isolated by Isopaque-Ficoll (Tianjin HY Bioscience Co., Ltd., Tianjin, China) gradient centrifugation. These cells were washed three times with PBS and further cultured in complete RPMI-1640 medium.

### Primary MM Cells

Primary human MM cells were recovered from fresh BM aspirates of MM patients. The hPBMCs were isolated by Ficoll density gradient centrifugation. CD138 positive cells were selected by magnetic bead sorting using the MidiMACS system (Miltenyi Biotec Inc., Bergisch Gladbach, Germany) according to the manufacturer’s instructions.

### Mice, Tumor Challenge, and Treatment

The animal study proposal and protocol were approved by the Second Hospital of Shandong University of Medicine Institutional Animal Care & Use Committee (approval no. KYLL-2017(LW)016). All experimental procedures involving animals were conducted in accordance with the experimental animal guidelines approved by the State Science and Technology Commission, PR China. Female Nod/LtSz-Prkdcscid/Prkdcscid (NOD/SCID) mice (6-8 weeks old) were obtained from the Beijing Vital River Laboratory Animal Technology Co., Ltd (Beijing, China) and kept under specific pathogen-free conditions. In order to evaluate local tumor growth, 1 × 10^7^ RPMI8226 or RPMI8226-Luc cells were subcutaneously injected (s.c.) into the right flank of NOD/SCID mice. The MM-implanted mice were then randomly divided into three groups (n = 6/group): the PBS control, exNK control, and exNK+Tim-3 blockage group. The mice received NK cells (1 × 10^8^) intravenously on days 7 and 14 after tumor cell inoculation. The tumor growth was assessed every 3 days by measuring tumor volume, calculated as V = lw^2^/2, where l = length and w = width. Four weeks later, the mice were sacrificed, and tumors were weighed. Tumor progression was measured at days 30 after tumor injection using an *in vivo* bioluminescence imaging system (IVIS Spectrum, PerkinElmer) according to manufacturer’s instructions. Also, the survival curve of mice i.v. injected with 2×10^6^ RPMI8226 cells (200μl) and mouse survival was evaluated twice a day.

### Flow Cytometry Analysis

For cell surface molecule staining, cells were harvested and stained with the labeled mAbs at 4°C for 45 min. For intracellular protein staining, cells were cultured in RPMI 1640 containing 10% FBS, and treated with monensin (Sigma) for 4 h to inhibit the extracellular secretion of cytokines. The antibodies used were as followed: FITC-conjugated Ab to CD3 and Granzyme B (R&D System), FITC-conjugated Ab to perforin (eBioscience, San Diego, CA, USA), PE-conjugated Ab to CD56, CD107a, Tim-3, Galectin-9, HMGB1, CEACAM1 (R&D Systems), or FITC/PE-conjugated anti-human IgG (eBioscience, San Diego, CA, USA). All stained cells were analyzed using a flow cytometer (Aria II, BD, USA), and the data were processed with Flowjo10.1 software (Scripps Research Institute).

### Cytokine ELISA

NK cells (2 × 10^5^ cells/well) were plated in triplicate 12-well plates with or without Tim-3 blockade for 48h. TNF-α and IFN-γ levels in cell culture supernatants were evaluated by commercial enzyme-linked immunosorbent assay (ELISA) kits (R&D Systems) according to the manufacturer’s instructions.

### Cytotoxicity Assay

NK cell-mediated cytotoxicity was determined by using PKH26 and Annexin-V staining. Annexin-V (Roche, Manheim, Germany) was used to detect apoptotic cells induced by NK cells. Target MM cells were stained with PKH26 dye (Sigma, St. Louis, USA) according to the protocol provided by the manufacturer. PKH26 stained target cells were co-cultured with NK cells at various Effector (E): Target (T) ratios for 4h. Then, the co-cultured cells were harvested and stained with Annexin-V. Cytotoxicity (%) = (Annexin-V^+^PKH26^+^ cells/PKH26^+^ cells) × 100%.

### Statistical Analysis

All data are expressed as the mean ± SD from 3 independent experiments. Statistical analysis was performed using the paired Student’s *t*-test, and the statistically significant differences were set at *p < 0.05, **p < 0.01. Statistical differences for mouse survival were analyzed using the Mann-Whitney *U* test.

## Results

### Tim-3 Expression in NK Cells and MM Cells

**Figure 1:**
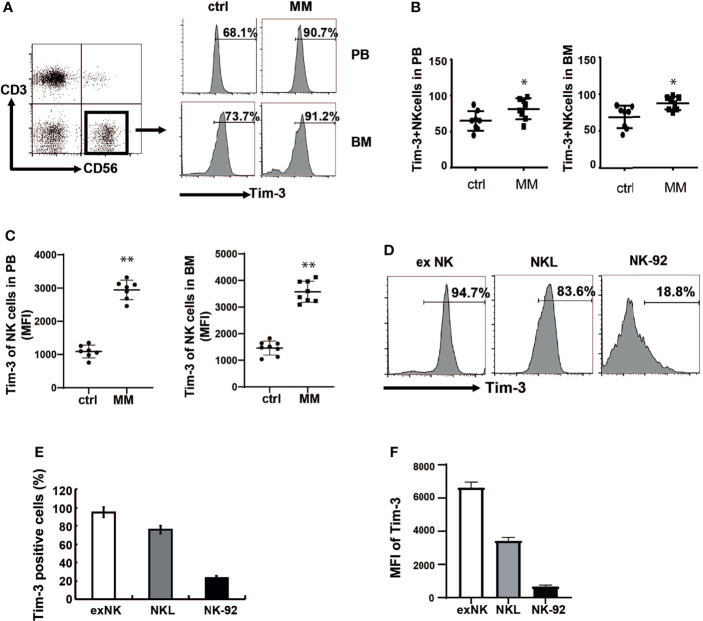
Tim-3 expression in primary NK cells and NK cell lines. Tim-3 expression was quantified by flow cytometry. **(A)** Tim-3 expression in the BM and peripheral blood NK cells of MM patients and control donors. Freshly isolated hPBMCs and hBMMCs were stained with CD3, CD56, Tim-3, and analyzed for the proportion of Tim-3^+^NK cells. **(B, C)** Statistical analysis of the proportion of Tim-3^+^NK cells and the MFI of Tim-3 in NK cells (n=8). **(D–F)** The expression of Tim-3 in exNK, NKL and NK-92 cells. One representative results from at least three independent experiments were presented. Data shown were mean ± SD of at least three independent experiments. *P < 0.05, **P < 0.01 versus control NK cells.

Expressions of Tim-3 in the BM and peripheral blood NK cells were analyzed from both MM patients and controls. at Tim-3 expressions of NK cells in the BM (**Figure 1A**, *P*<0.02) and peripheral blood (**Figure 1B**, *P*<0.05) from MM patients was significantly increased compared to those from the control groups.

Then expressions of Tim-3 in ex NK cells and two NK cell lines (NK-92 and NKL cells) were analyzed. ex NK cells were derived from the peripheral blood of healthy donors, according to our previous research method. After 21 days of expansion, the number of cells increased by about 1000 times, and the purity of NK cells reached a peak value of 85% (data not shown). As shown in **Figures 1D**–**F**, Tim-3 was expressed in ex NK cells and the two NK cell lines. Among the three NK cells, Tim-3 had the highest expression in exNK cells, about 95%.

Tim-3 could also be expressed on some MM cells. The expression ratio was 67% in RPMI8226 cells, while the expression level in MM.1s cells was relatively low (**Supplementary Figure S2**).

### Tim-3 Ligand Expression in MM Cells and NK Cells

**Figure 2:**
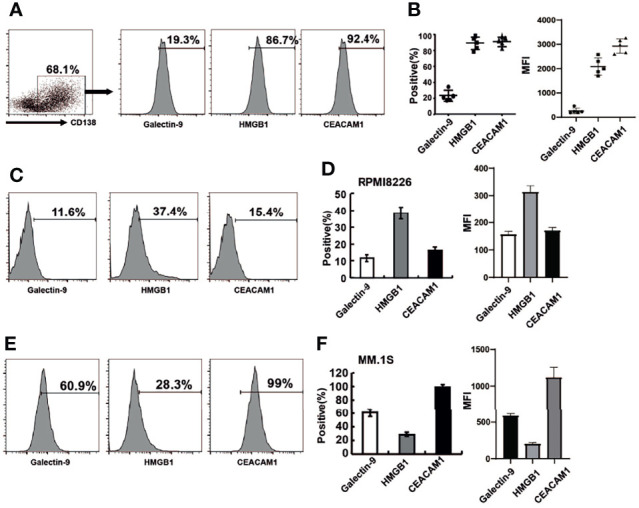
The expression of Tim-3 ligands in primary MM cells and MM cell lines. Tim-3 ligands expression were quantified by flow cytometry. **(A)** CD138^+^ MM cells were sorted by immunomagnetic separation from the bone marrow of MM patients and stained with Galetin-9, HMGB1, CEACAM1 for the proportion of Tim-3 ligands^+^MM cells. **(B)** Statistical analysis of the proportion and MFI of Tim-3 ligands^+^MM cells (n=5). **(C, D)** The expression of Tim-3 ligands in MM cell line RPMI8226 cells. **(E, F)** The expression of Tim-3 ligands in MM cell line MM.1S cells. Representative results from one of at least three independent experiments were exhibited. Data shown were mean ± SD of at least three independent experiments.

When Tim-3 binds to its ligands, the function of NK cells is inhibited, resulting in reduced secreting cytokine secreting and target cell killing. The homologous ligands of TIM-3 include galectin-9, HMGB1, and CEACAM-1. All three Tim-3 ligands were expressed in primary MM cells as shown in **Figures 2A, B**. Among them, the level of HMGB1 and CEACAM-1 expression was as high as 90%, while galectin-9 was expressed in approximately 20% of cells.

Tim-3 ligand expression was also analyzed in MM cell lines RPMI8226 and MM.1S. The levels of Tim-3 ligands expression in RPMI8226 for galectin-9, HMGB1 and CEACAM-1 were 11.6%, 37.4%, and 15.4%, respectively. HMGB1 was dominantly expressed in RPMI8226, while CEACAM-1 was dominantly expressed in MM.1S, indicating differences in Tim-3 ligand usage in different MM cell lines or cells (**Figures 2C–F**).

Most Tim-3 ligands were not expressed on NK cells, only CEACAM1 was expressed on NK-92 cells (70%).

### Tim-3 Blockade Enhanced NK Cell- Mediated Natural Cytotoxicity Against Human MM Cells

**Figure 3:**
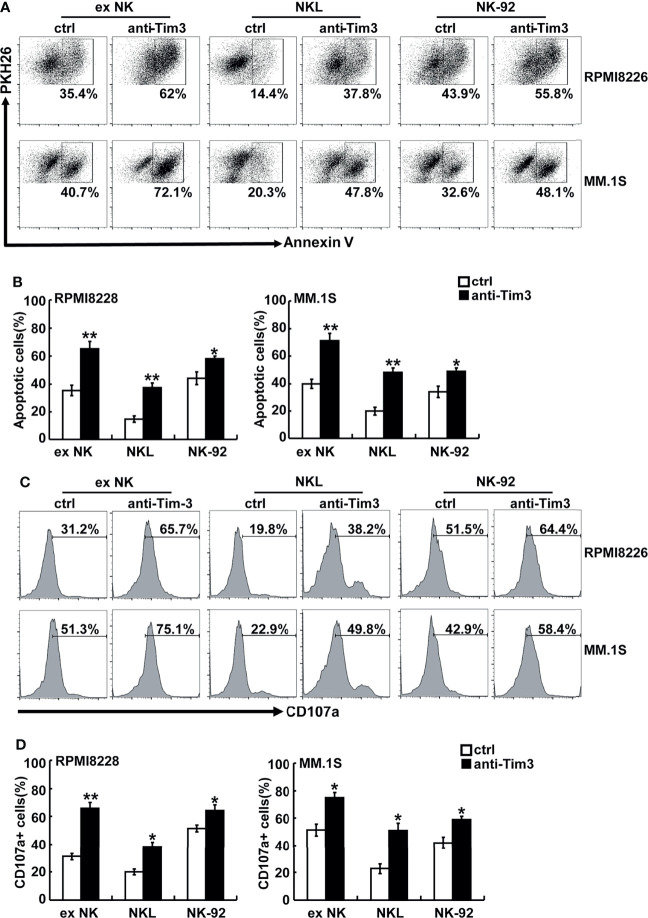
Tim-3 blockade enhanced NK cell-mediated natural cytotoxicity against human MM cell lines. exNK, NKL, NK-92 cells were used as effector (E) cells and human MM cell lines were used as target (T) cells. The E:T ratios of exNK, NKL, NK-92 cells were 1:1, 5:1, 1:1, respectively. **(A, B)** Cytotoxicity activity of NK cells was tested by PKH26/Annexin-V stain. The cytotoxicity of NK cells was determined by the percentage of apoptotic MM cells at the indicated E:T ratios after pre-incubation with 10 μg/ml anti-Tim-3 antibodies. **(C, D)** The degranulation level of NK cells was detected by CD107 degranulation assay. The dot plots illustrating CD107a surface expression on the NK cells after stimulation by the target cell lines, respectively. One representative of at least three independent experiments. Data shown were mean ± SD of at least three independent experiments. *P < 0.05, **P < 0.01 versus control NK cells.

Anti-Tim-3 antibody was used to block the Tim-3 pathway of NK cells. To test the killing ability of NK cells, MM cells were pre-stained with PKH26 and then incubated with NK cells. The apoptotic MM cells (PKH26+Annexin-V+) were quantified using flow cytometry. **Figures 3A, B** show that NK cells significantly induced apoptosis of RPMI8226 and MM.1S cells. The killing activity of exNK cells and NKL cells to the two MM cell lines increased by about 30% and 20%, respectively, compared to the control group. The surface translocation assay of CD107a was used to determine the degranulation activity of NK cells (**Figures 3C, D**). Generally, the level of NK cell degranulation activity represents their killing ability. Our results showed that after Tim-3 blockade, degranulation activity of exNK cells, NK-92 cells and NKL cells were all significant augmented.

**Figure 4:**
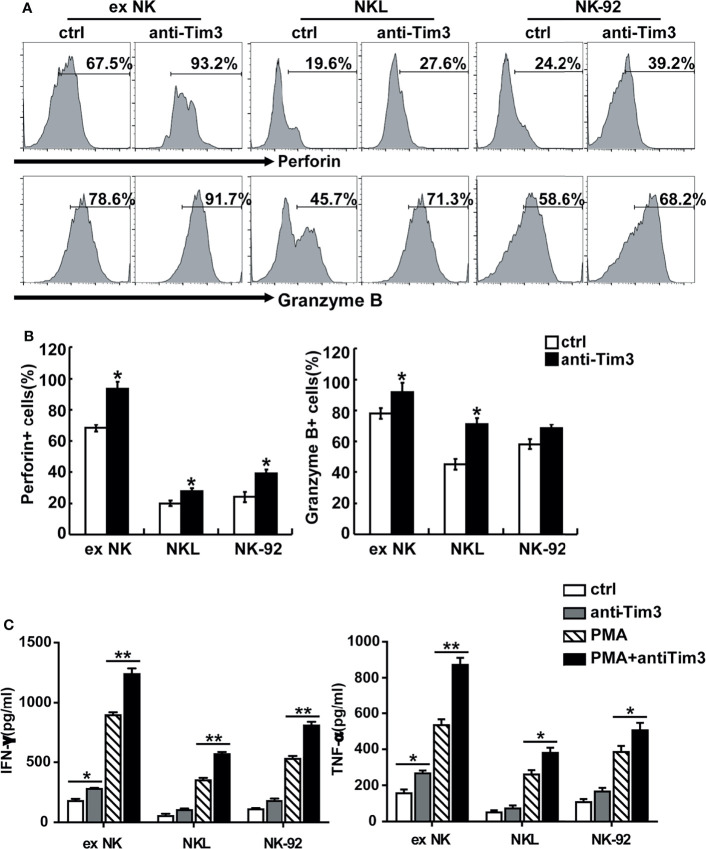
Tim-3 blockade enhanced perforin and Granzyme B expression as well as cytokine secretion in NK cells. **(A, B)** exNK, NKL, NK-92 cells were pre-incubated with 10 μg/ml anti-Tim-3 or isotype antibodies for 48h. The protein levels of perforin and Granzyme B in NK cells were analyzed by flow cytometry. One representative of at least three independent experiments. Data shown were mean ± SD of at least three independent experiments. *P < 0.05, **P < 0.01 versus control NK cells. **(C)** exNK, NKL, NK-92 cells were pre-incubated with 10 μg/ml anti-Tim-3 or isotype antibodies. Production of TNF-α and IFN-γ by NK cells after stimulation with/no PMA/ionomycin for 48 h, as evaluated by ELISA assay. Data shown were mean ± SD of at least three independent experiments. *P < 0.05, **P < 0.01 versus control NK cells.

The activation and cytotoxicity of NK cells are affected by expression of cytotoxicity-related molecules, including NKG2D, NKG2A, TNF-α, IFN-γ, perforin, granzyme B, and Fas ligand (FasL). All of these cytotoxicity-related molecules were detected and differences in perforin, granzyme B, IFN-γ, and TNF-α were found. The protein levels of perforin and granzyme B were determined using FACS. As shown in **Figures 4A, B**, blocking Tim-3 significantly increased expression of perforin and granzyme B in NK cells. The secretion levels of TNF-α and IFN-γ were measured by ELISA. As shown in **Figure 4C**, after Tim-3 blockade, NK cells expressed higher levels of IFN-γ than control NK cells. After stimulation with PMA/ionomycin for 48 hours, the concentration of IFN-γ secreted by exNK cells increased from 850 to 1200 pg/ml when treated with the Tim-3 antibody.

**Figure 5:**
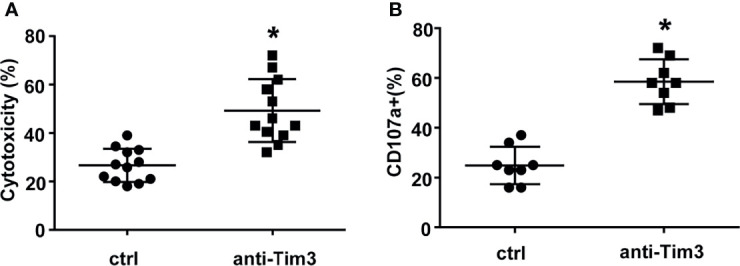
Tim-3 blockade enhanced NK cell-mediated natural cytotoxicity against primary human MM cells. Human primary MM cells from 12 relapsed/refractory MM patients and 11 control donors were used as target (T) cells. CD138^+^ MM cells were sorted by immunomagnetic separation from the bone marrow of MM patients. exNK cells were used as effector (E) cells and the E:T ratios was 1:1. Cytotoxicity activity of NK cells was tested by PKH26/Annexin-V stain. *P < 0.05 versus control NK cells. Cytotoxicity activity **(A)** and CD107a surface expression **(B)** of NK cells were tested.

Subsequently, we evaluated the cytotoxicity of NK cells against primary MM cells isolated from 12 relapsed/refractory MM patients. Compared to control NK cells, NK cells with Tim-3 blockade displayed enhanced cytotoxicity towards MM cells (**Figure 5A**, *P*<0.05). The degranulation of NK cells markedly increased after Tim-3 blockage (**Figure 5B**, *P*<0.05). These results indicated that blocking Tim-3 could effectively enhance the cytotoxicity of NK cells to MM cells.

### The Role of Tim-3 Ligands in the Killing Process of NK Cells

**Figure 6:**
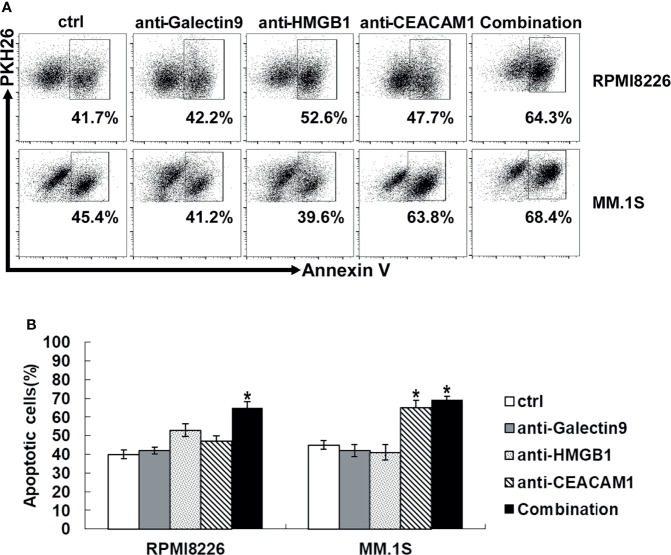
Tim-3 ligands combined blockade enhanced NK cell-mediated natural cytotoxicity against human MM cell lines. exNK was used as effector (E) cells and human MM cell lines were used as target (T) cells. MM cell lines were pre-incubated by 10 μg/ml anti-Tim-3 ligands antibody. Cytotoxicity activity of NK cells was tested by PKH26/Annexin-V stain. *P < 0.05 versus control NK cells. **(A)** Cytotoxicity activity of NK cells. **(B)** Statistical Analysis of **(A)**.

When Tim-3 on NK cells bind to its ligands, the NK cell function is inhibited. All three Tim-3 ligands were expressed on primary MM cells and MM cell lines, whileHMGB1 and CEACAM1 had a high level of expression, as described above. In order to explore the key molecules that mediated the cytotoxicity of NK cells, the blocking antibodies of the three Tim-3 ligands were selected for evaluation. As shown in **Figure 6**, when the Tim-3 ligands on MM cells were blocked, the killing level of NK cells increased to varying degrees. Especially when CEACAM1 in MM.1S cells were blocked, the killing function of NK cells improved robustly. When the Tim-3 ligands on MM cells were blocked in combination, exNK cells had much stronger cytotoxic ability.

### Tim-3 Blockade Substantially Improved the Anti-MM Effects of NK Cells *In Vivo*

**Figure 7:**
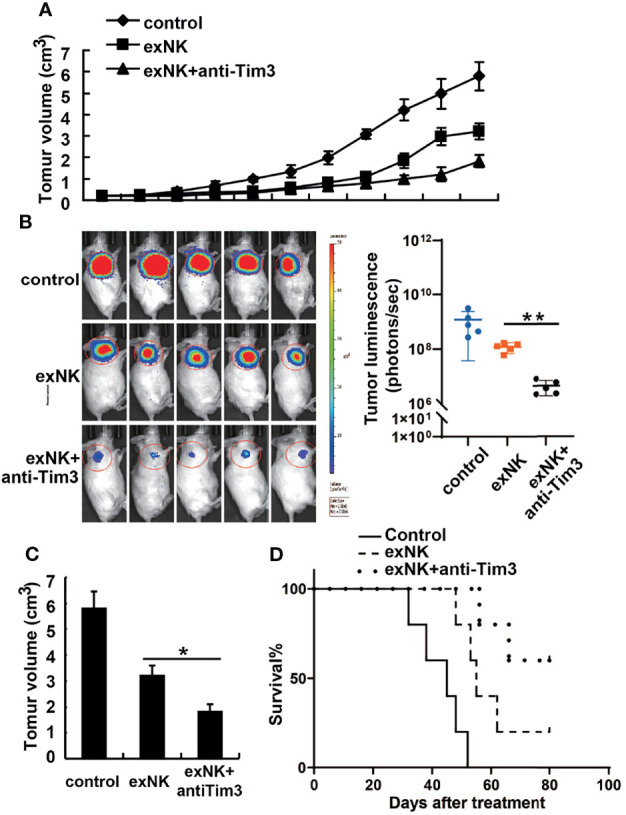
Anti-tumor effects of Tim-3 blockade NK cells on human MM tumor-bearing NOD/SCID mice. **(A–C)** Female NOD/SCID mice were subcutaneously injected with 1×10^7^ RPMI8226 cells and then intravenously injected with 1×10^8^ exNK cells with anti-Tim3 or isotype antibodies on day 7 and 14 post of tumor cell inoculation. The tumor volumes of tumor-bearing mice were measured every 3 days. The tumor burden was monitored by bioluminescence imaging with the IVIS system and the total tumor luminescence (photons/sec) of the tumors were measured. **(D)** NOD/SCID mice were i.v. injected with 2×10^6^ RPMI8226 cells and then injected intravenously with 2×10^7^ exNK cells with anti-Tim3 or isotype antibodies on day 7 and 14 post of tumor cell inoculation. The curves for the survival were shown as percentages of the initial number of animals per group. *P < 0.05, **P < 0.01 versus control NK cells.

The *in vivo* anti-MM effect of NK cells was evaluated on MM-cell bearing mice. First, mice were subcutaneously injected with 1 × 10^7^ RPMI8226 or RPMI8226-Luc cells. When the tumor models were established, the mice were randomly assigned into three groups, and treated with PBS, control exNK cells, and exNK + anti-Tim3 cells, respectively, on day 7 and 14 post of inoculation. As depicted in **Figure 7A, C**, exNK + anti-Tim3 cells exerted a significantly strong inhibitory effect on RPMI8226 cells compared to control exNK cells. After approximately 30 days, the tumor volumes in the PBS control group and control exNK group were 346 ± 26 mm^3^ and 198 ± 27 mm^3^, respectively, whereas the tumor volumes in the exNK + anti-Tim3 group was only 37 ± 11 mm^3^, showing an 81.3% inhibition ratio (p < 0.01, compared with the control exNK group). The tumor burden was monitored by bioluminescence imaging with the IVIS system. As shown in **Figure 7B**, Total tumor luminescence (photons/sec) of the exNK + anti-Tim3 group was significantly lower to control exNK group (p < 0.01, compared with the control exNK group). RPMI8226 cells were further i.v. injected into NOD/SCID mice and they then received treatments as above. Kaplan–Meier curves (**Figure 7D**) showed that the exNK + anti-Tim3 cells treatment significantly prolonged the survival of MM cell-bearing mice (80 *vs* 50 days, *P* < 0.05). These data indicated that NK cells with Tim-3 blockade inhibited the growth and development of MM in the early and late stages of the disease.

## Discussion

NK cells are an important part of innate immunity and can directly clear infected cells and tumor cells earlier than T cells, without pre-sensitization and activation. In anti-tumor immunity, NK cells selectively recognize “non-self” tumor cells that have low expression of MHC-I molecules missing, inhibit tumor growth and metastasis to other organs, and effectively eliminate tumors (17, 18). In early studies, NK cell-based adoptive immunotherapy mainly obtained NK cells by removing leukocytes and magnetic bead sorting. These methods were costly, and the process was complicated, but the number of NK cells obtained was very limited (19). The anti-CD16 antibody and IL-2 amplified exNK cells used in this study have high purity and strong activity, which can be produced on a large scale to meet the needs of clinical treatment (9). In addition, this study also considered the possibility of NK cell lines for treatment, including NK-92 and NKL cell lines. Among them, NK-92 cells are the first NK cell line used in clinical trials. Therefore, we used exNK cells, NK92, and NKL cell lines in our study in order to provide support data for NK cell-mediated adaptive immunotherapy.

PD-1 and Tim-3 are both immune checkpoint molecules and markers of immune cell exhaustion (20). Many clinical studies have shown that the PD-1 antibody is effective in treating melanoma, lung cancer, lymphoma, and other tumors (21). However, the treatment of MM with the anti-PD-1 antibody alone did not show a therapeutic effect, and the phase I clinical trial failed (22). Tim-3 is likely to be an important negative regulator of NK cells with an anti-myeloma effect. Our results indicate that both MM cell lines and primary MM cells derived from MM patients express Tim-3 ligands, suggesting that the MM bone marrow microenvironment is involved in expression of Tim-3 in NK cells and Tim-3 ligands in MM cells. The application of antibodies to block Tim-3 can significantly improve the killing of myeloma cells by NK cells. These findings suggest that the Tim-3/ligands pathway may play an important role in helping MM cells escape from immune surveillance. The effect of blocking the Tim-3/ligands pathway in the treatment of MM is worth studying in depth.

Tim-3 blockade significantly augmented the natural cytotoxicity of NK cells towards the human MM cell lines RPMI8226 and MM.1S, as well as primary human MM cells isolated from relapsed/refractory MM patients. Interestingly, due to the different expression levels of Tim-3 in the three kinds of NK cells, Tim-3 blockade has different effects on NK cell killing. Among them, the Tim-3 expression level in exNK cells was the highest, and the Tim-3 antibody blockade also had the strongest effect in exNK cells, indicating that the Tim-3 expression level was positively correlated with its blocking effect. Patients with relapsed/refractory MM usually have very limited clinical treatment options as well as poor therapeutic outcomes. Our data indicate that Tim-3 blockade in NK cells might be used as an alternative option to the treatment of MM.

Tim-3 is a key immune checkpoint molecule for tumor-induced immune suppression. When Tim-3 on NK cells bind to its ligands, its function of secreting cytokines and killing target cells is diminished (20–22). It has been assumed that the Tim-3-Galectin-9 pathway plays an important role in regulating tumor cells escaping from immune surveillance (23, 24). In our study, of all the Tim-3 ligands, only CEACAM1 was expressed on NK-92 cells (about 70%). We believed that Tim-3 on NK cells mainly acted by binding to ligands on target cells. However, the expression level of Galectin-9 in both primary MM cells and MM cell lines was low. Antibody blocking experiments further confirmed that HMGB1 and CEACAM1 play more important roles in the system of NK cells killing MM cells. Therefore, the Tim-3- HMGB1/CEACAM1 rather than Tim3-Galectin-9 pathway is the major player in MM evasion of NK cell mediated cytotoxicity.

Furthermore, we demonstrated that Tim-3 blocked NK cells could significantly enhance survival and inhibit tumor growth in MM tumor-bearing mice. As Tim-3 blocking NK cells increased the survival of mice bearing MM tumors by approximately 30% compared to mice injected with control NK cells, the adoptive transfer procedure could be further optimized. For example, the number of injections or the number of NK cells injected could be increased. Overall, these results indicate that Tim-3 blockade enhances the natural cytotoxicity of NK cells towards MM cells both *in vitro* and *in vivo*.

Tim-3, which has multiple ligands, is widely present in many types of immune cells. Tim-3 inhibits tumor immunity by inducing Th1 cell apoptosis and NK, CD8+T cell depletion. Therefore, the cellular immune response could be regulated by blocking the Tim-3 signaling pathway, thereby reducing or preventing the development of tumors and improving the prognosis. Tim-3 is highly expressed in immune cells of liver, lung, breast, gastric and cervical cancer, and other solid tumors, and its expression intensity is also related to the progression and grade of hematological malignancies (25–31). The latest research shows that Tim-3 blocking antibody therapy has entered phase Ia/b clinical research in advanced solid tumor patients (32–34).

## Conclusion

Our results presented in the current study show that Tim-3 blockage significantly enhances primary NK cells and NK cell line mediated-killing of MM cells *in vitro* and *in vivo*, supporting the notion that Tim-3 may be an important target molecule used for developing an antibody and/or NK cell based immunotherapeutic strategies for MM.

## Data Availability Statement

The original contributions presented in the study are included in the article/**Supplementary Material**. Further inquiries can be directed to the corresponding authors.

## Ethics Statement

The studies involving human participants were reviewed and approved by the Human Ethics Research Committee of the Second Hospital of Shandong University (approval no. KYLL-2017(KJ) P-0106). The patients/participants provided their written informed consent to participate in this study. The animal study was reviewed and approved by the Second Hospital of Shandong University of Medicine Institutional Animal Care & Use Committee (approval no. KYLL-2017(LW)016).

## Author Contributions

CZ and XF designed the study, performed research, and analyzed data. WJ, FL, YJ, SL, XL, and YX performed research and analyzed data. WJ, XF, and CZ wrote the manuscript. All authors contributed to the article and approved the submitted version.

## Funding

This work was supported by the National Natural Science Foundation of China (No.81600176), the Natural Science Foundation of Shandong Province (No.ZR2016HB71;ZR2020MH123), the Science and Technology innovation project of Shandong Province (No.2017GSF18136 and No.2018GSF118034), Key Research and Development Program of Shandong Province (No. 2021CXGC011101) and Rongxiang Regenerative Medicine Foundation of Shandong University (2019SDRX-05).

## Conflict of Interest

Author BL was employed by Weihai Zhengsheng Biotechnology Co., Ltd.

The remaining authors declare that the research was conducted in the absence of any commercial or financial relationships that could be construed as a potential conflict of interest.

## Publisher’s Note

All claims expressed in this article are solely those of the authors and do not necessarily represent those of their affiliated organizations, or those of the publisher, the editors and the reviewers. Any product that may be evaluated in this article, or claim that may be made by its manufacturer, is not guaranteed or endorsed by the publisher.

## Supplementary Material

Supplementary Figure S1 The expression of Tim-3 ligands in exNK cells and NK cell lines. Tim-3 ligands expression were quantified by flow cytometry. **(A-C)** The expression of Tim-3 ligands in exNK, NK-92 and NKL cells.

Supplementary Figure S2 Tim-3 expression in MM cell lines. Tim-3 expression were quantified by flow cytometry. Figure showed the expression of Tim-3 ligands in MM cell line RPMI8226 and MM.1S cells.
