# Supplementary figures and images for: Tim-3 Blockade Elicits Potent Anti-Multiple Myeloma Immunity of Natural Killer Cells
Source: Front Oncol. 2022 Feb 25;12:739976. doi: 10.3389/fonc.2022.739976 (PMC8913933; doi:10.3389/fonc.2022.739976)

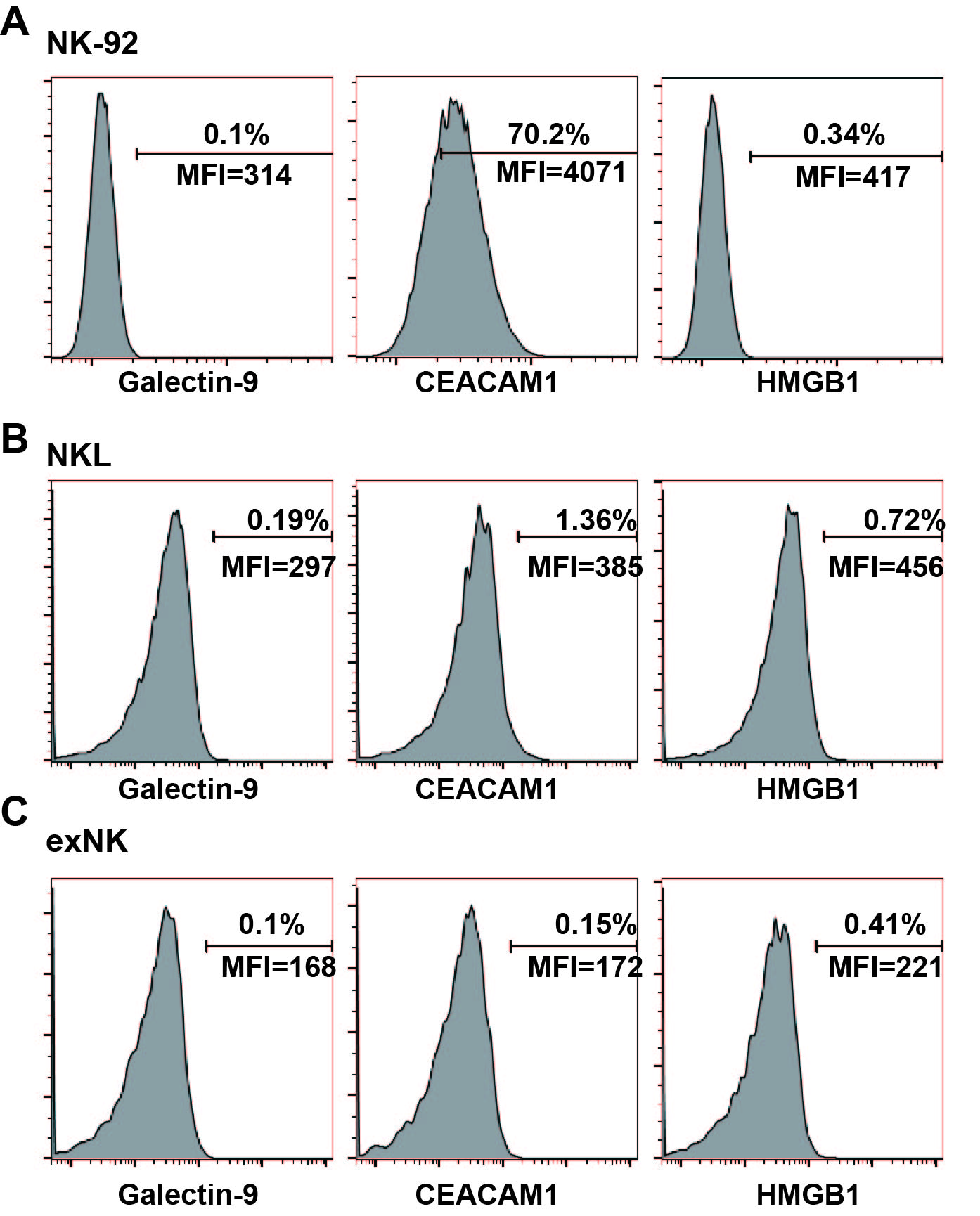

Supplement: Supplementary Figure S1 — The expression of Tim-3 ligands in exNK cells and NK cell lines. Tim-3 ligands expression were quantified by flow cytometry. (A-C) The expression of Tim-3 ligands in exNK, NK-92 and NKL cells. [file Image_1.jpeg]

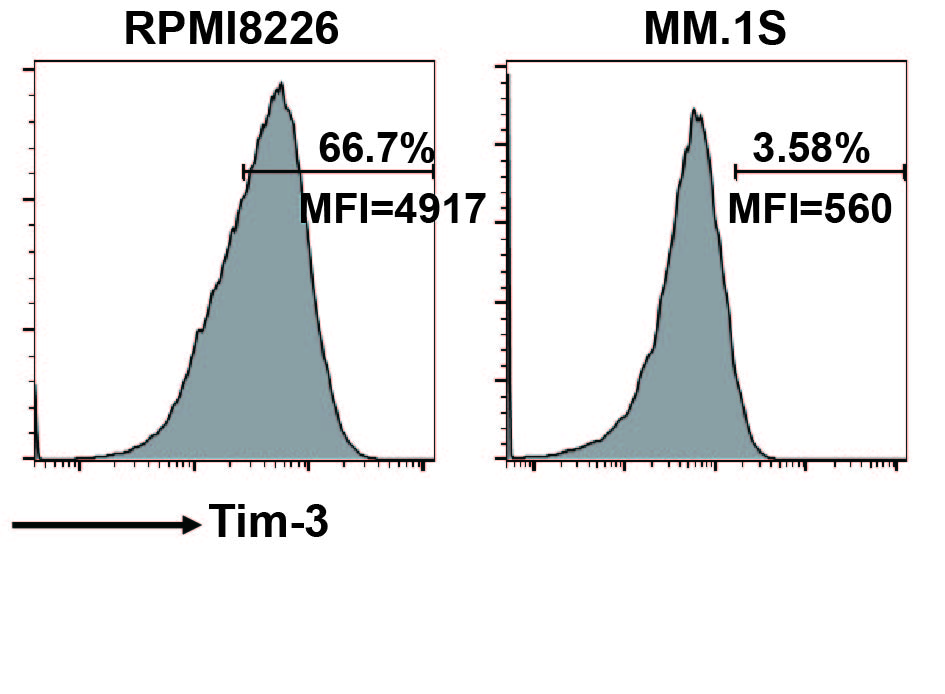

Supplement: Supplementary Figure S2 — Tim-3 expression in MM cell lines. Tim-3 expression were quantified by flow cytometry. Figure showed the expression of Tim-3 ligands in MM cell line RPMI8226 and MM.1S cells. [file Image_2.jpeg]
